# The clinical and epidemiological landscape of *Mycobacterium riyadhense*: a systematic review of the literature

**DOI:** 10.3389/fcimb.2026.1761754

**Published:** 2026-04-13

**Authors:** Hassan Almarhabi, Abdulmajeed Sarhan, Abdulellah Almohaya, Aisha Alharbi

**Affiliations:** 1 Department of Medicine, King Abdulaziz Medical City, Ministry of National Guard Health Affairs, Jeddah, Saudi Arabia; 2 King Abdullah International Medical Research Centre, Jeddah, Saudi Arabia; 3 King Saud Bin Abdulaziz University for Health Sciences, Jeddah, Saudi Arabia; 4 Department of Medicine, Ministry of National Guard - Health Affairs, Riyadh, Saudi Arabia; 5 King Abdullah International Medical Research Center, Riyadh, Saudi Arabia; 6 King Saud Bin Abdulaziz University for Health Sciences, Riyadh, Saudi Arabia; 7 Pharmaceutical Care Department, King Abdulaziz Medical City, Ministry of National Guard Health Affairs, Jeddah, Saudi Arabia

**Keywords:** antimicrobial sensitivity tests, clinical presentations, *Mycobacterium riyadhense*, outcome, risk factors, systematic review, treatment

## Abstract

**Background:**

*Mycobacterium riyadhense* is an emerging nontuberculous mycobacterium associated with infections in immunocompetent and immunocompromised patients.

**Methods:**

To identify the epidemiology, risk factors, presentations, and antimicrobial susceptibility of *M. riyadhense*, we conducted a systematic review on PubMed, Web of Science, and Google Scholar adhering to PRISMA guidelines. We included publications during 2009-2025, with no limitation on language or study design. Cases of uncertain infection or identification were excluded. Review protocol was pre-registere in PROSPERO with protocol number; CRD420250653025.

**Results:**

We identified 22 studies reporting on 69 cases of *M. riyadhense*. Patient median age was 35.5 and 80% were male. Common presentations included pneumonia (n=52), lymphadenitis (n=13), osteomyelitis (n=8), or disseminated infection (n=8). Susceptibility to Rifampin and Ethambutol was observed in all tested isolates. Resistance to Isoniazid was detected in 5/8 isolates.

**Conclusion:**

This review identified a significant variability in the diagnosis and treatment of *M. riyadhense*. This underscores the urgent need for standardized diagnostic protocols and targeted treatment strategies to effectively manage infections.

**Systematic review registration:**

https://www.crd.york.ac.uk/prospero/, identifier CRD420250653025.

## 1 Introduction

*Mycobacterium riyadhense (M. riyadhense)*, is an emerging global pathogen that is increasingly recognized as a cause of infection in both immunocompetent and immunocompromised patients (Sawan et al; van Ingen et al., 2009; Choi et al., 2012; Godreuil et al., 2012; Henderson et al., 2012; Garbati and Hakawi, 2014; Saad et al., 2015; Al-Ammari et al., 2016; Al-Dossary et al., 2016; Alenazi et al., 2019; Sapriel and Brosch, 2019; Varghese et al., 2019; Baadani et al., 2020; Almutairi et al., 2022; Kosaraju et al., 2024; Almarhabi et al., 2025). It is a slow-growing, non-chromogenic, nontuberculous mycobacterium (NTM) that belongs to the *Mycobacterium tuberculosis* (MTB) associated clade, the group most closely related to MTB species complex (Sapriel and Brosch, 2019). It was identified in 2009 from the maxillary sinus of a Saudi patient and was named after the city of Riyadh, Saudi Arabia (van Ingen et al., 2009). However, retrospective analyses of previously misidentified NTM isolates revealed earlier clinical occurrences of *M. riyadhense* in France, 2005 (Godreuil et al., 2012), and Bahrain, 2006 (Godreuil et al., 2012). Since then, additional cases have been reported worldwide (Sawan et al; Choi et al., 2012; Henderson et al., 2012; Varghese et al., 2017; Xu et al., 2017; Varghese et al., 2018). *M. riyadhense* appears to be environmentally ubiquitous, with documented isolation from water samples in Ethiopia, Sweden, and the United States (Londono Zuluaga, 2016; Hongslo and Jansson, 2016; King et al., 2017), soil samples in Canada and Malaysia (Maidin et al., 2016; Narendrula-Kotha and Nkongolo, 2017), plumbing systems in Japan (Rahmatika et al., 2022), and tobacco products imported to Saudi Arabia (Alqumber, 2021). Accurate identification of *M. riyadhense* remains challenging as it requires advanced techniques, such as targeted sequencing, metagenomics, or mass spectrometry to distinguish it from other mycobacterial species (Almarhabi et al., 2025). Moreover, due to the lack of standardized susceptibility testing methods and established clinical breakpoints, the optimal therapeutic regimen remains unclear, underscoring the need for evidence-based guidance. This systematic review aims to summarize the current evidence on *M. riyadhense*’s epidemiology, disease presentations, antimicrobial susceptibility, treatment options, and clinical outcomes.

## 2 Methods

### 2.1 Search strategy

A comprehensive literature search was conducted using PubMed, Web of Science, and Google Scholar. The search terms “*riyadhense*” OR “*M. riyadhense*” OR “*mycobacterium riyadhense*” were used with no filters. We included all articles published from 2009 to November 16th, 2025, with no language or study design restrictions. Reference lists of all included articles were reviewed. Studies were included if they reported original clinical cases of *M. riyadhense*. According to the population, issue, outcome (PIO) framework, this review focused on the population of all patients infected with *M. riyadhense*, the issue concerned the collection of all clinically relevant data including disease presentations, antimicrobial susceptibility, and treatment options. The primary outcome was the success of antimicrobial therapy, defined as the complete resolution of clinical symptoms with no recurrence during treatment or follow-up.

### 2.2 Eligibility criteria

To be classified as true infection, cases were required to demonstrate clinical signs and symptoms consistent with an infectious process and to have microbiological confirmation of *M. riyadhense* through culture or genetic sequencing from affected anatomical sites. Exclusion criteria included insufficient evidence to distinguish infection from colonization, cases in which M. tuberculosis or other pathogenic NTM could not be ruled out as primary pathogens, and cases where the identification of *M. riyadhense* was not certain.

### 2.3 Data collection

Full-text review was conducted by two authors (A.S, A.A) with no automation tools. Discrepancies were resolved through discussion with a third author (H.A). Data extraction was performed manually and entered into prestructured Microsoft Word tables.

### 2.4 Quality assessment

The quality of each article was assessed using standardized critical appraisal tools published by the Joanna Briggs Institute (JBI, University of Adelaide, Australia). Articles were given a score representing the number of items reported adequately out of total items required in the appraisal checklist for the specific article type, i.e., case report or series. The max score was 8/8 for case reports and 10/10 for case series.

### 2.5 Data analysis

Patients aged 65 years or older were categorized as elderly, while those younger than 18 were classified as pediatrics. Disseminated infection was defined as the involvement of two or more non-contagious organs (Abukhalid et al., 2025). Numerical values were presented as percentage (n/total) or median (Q1 – Q3), as appropriate. All studies meeting inclusion criteria were included in the presented data. Missing information was requested through email; however, only one author responded out of 6 contacted. Information obtained through correspondence is presented in the **Supplementary Materials** (**Appendix A**) (Almutairi et al., 2022). The denominator in each data point represents the total reported cases for which the respective information is available without any assumptions (see **Tables 1**, **2**). The review protocol was pre-registered in PROSPERO under protocol number; CRD420250653025.

**Table 1:** Characteristics of studies included in the review; 22 articles reporting on 69 cases.

Study reference	Cases	F/M ratio	Age; median (IQR)	Risk factors	Pulmonary	Lymphatic	Other	Failed regimens
Varghese 2017 (Varghese et al., 2017)	12	1/11	47 (26.5 - 69)	N/A	9	3	0	INH/RIF: 1*
Varghese 2018 (Varghese et al., 2018)	15	N/A	N/A	N/A	12	1	Bone (n= 2)	N/A
Almutairi 2022 (Almutairi et al., 2022) *Guan 2021* (Guan et al., 2021)	11	1/11	35 (28 – 58)	HIV: 5 Lymphoma: 1 Hx of TB: 1	10	2	Pleura (n= 1)	N/A
Varghese 2019 (Varghese et al., 2019)	2	0/2	8 (N/A)	CF: 1 CN: 1	1	1	0	N/A
Baadani 2024 (Baadani et al., 2020)	1	N/A	N/A	HIV: 1	N/A	N/A	N/A	N/A
Xu Z 2017 (Xu et al., 2017)	1	N/A	N/A	N/A	0	1	0	N/A
Case report/series data (**Table 2**)	27	10/17	36 (21.5 – 43.5)	HIV: 8 Gastric Sx: 2 Smoking: 2 SLE/APS: 1 SCID: 1 AR-IMD91: 1 Lymphoma: 1 Trauma: 1 Hx of TB: 1	20	5	Bone (n= 6) CNS (n= 1) Sinus (n= 1) Liver (n= 1) Joint (n= 1) GN (n= 1)	LFX: 1 INH/RIF: 2^a^ CLR/CIP: 1
Total	69	12/41	35.5 (22– 48)	HIV: 14 Other IMD: 3 Lymphoma: 2^b^ Hx of TB: 2^c^ Other: 8	52	13	Disseminated (n= 8) Bone (n= 8) Other (n= 6)	LFX: 1 INH/RIF: 3 CLR/CIP: 1

Details of all studies with reported *M. riyadhense* infection. Case reports with full case descriptions are reported in **Table 2**. F, Female; M, Male; HIV, Human Immunodeficiency Virus; Hx of TB, previous history of tuberculosis; CF, cystic fibrosis; CN, congenital neutropenia; Sx, surgery; SLE, systemic lupus erythematosus; APS, antiphospholipid syndrome; SCID, Severe combined immunodeficiency; AR-IMD91, Autosomal recessive immunodeficiency 91; GN, glomerulonephritis; Other IMD, immunodeficiency other than HIV; INH, isoniazid; RIF, rifampin; EMB, ethambutol; CLR, clarithromycin; CIP, ciprofloxacin; LFX, levofloxacin; N/A, Information not available.

All studies are from Saudi Arabia, except (Xu et al., 2017); China. Pooled data from **Table 2** represents cases from different countries.

*Patient died, however, it could not be confirmed if this was due to ongoing infection.

^a^ Two failures occurring in a single case.

^b^ cooccurring with HIV (n= 1) and SCID (n= 1).

^c^ cooccurring with HIV (n= 1).

**Table 2:** Details of *M. riyadhense* case reports/series data; 27 cases.

Case # reference	Organs affected	Age	Sex	Risk factors	Year	Regimen/duration in months		Status
1*^†^ Henderson 2012, USA (Henderson et al., 2012)	Lung	29	M	None	2012	LFX	3	Relapse
						INH, RIF, EMB, PZA	16	Cured
2* Godreuil 2012, France (Godreuil et al., 2012)	Lung	39	F	None	2005	INH, RIF, EMB, PZA	2	Cured
						INH, RIF	10	
3*^‡^ Godreuil 2012, Bahrain (Godreuil et al., 2012)	Lung	43	M	None	2006	CLR, CIP	12	Relapse
						INH, RIF, EMB, PZA, CLR, CIP	8	Cured
4* Van Ingen 2009, KSA (van Ingen et al., 2009)	Sinus, Bone, Optic nerve	19	M	Blunt Trauma	2009	INH, RIF, EMB	2	Cured
						INH, RIF	7	
5* Saad 2015, KSA (Saad et al., 2015)	Bone, Dura mater	18	F	None	2012	INH, RIF, EMB, PZA	2	Cured
						INH, RIF, EMB, MOX	7	
						RIF, EMB	6	
6* Saad 2015, KSA (Saad et al., 2015)	Bone	24	F	None	2012	INH, RIF, EMB, PZA	6	Cured
						RIF, EMB	7	
7* Choi 2012, Korea (Choi et al., 2012)	Lung	38	F	None	2012	INH, RIF, EMB, PZA	8	Cured
						RIF, EMB, PZA	6	
8*^§^ Sawan 2024, UAE (Sawan et al)	Lung	44	F	None	2021	INH, RIF, EMB, PZA	2	Relapse
						INH, RIF	4	
						INH, RIF, EMB, PZA	2	Relapse
						INH, RIF	3	
						EMB, PZA, MOX, LZD	1	Cured
						RIF, EMB, PZA, LZD	1	
						INH, RIF, EMB, PZA, CLR + Sx	N/A	
9* Alenazi 2019, KSA (Alenazi et al., 2019)	Lung	44	F	HIV	2013	EMB, MOX, AZM	12	Cured
10* Alenazi 2019, KSA (Alenazi et al., 2019)	Lung	51	M	HIV	2015	MOX, CLR	10	Cured
11* Alsaeed 2025, KSA (Alsaeed et al., 2025)	Lymphatic	19	F	Morbid obesity, Post-gastric sleeve	2019	RIF, EMB, CLR RIF, MOX	N/A	Cured
12* Alsaeed 2025, KSA (Alsaeed et al., 2025)	Lung	47	M	HIV	2019	RIF, EMB, CLR EMB, CLR	N/A	Cured
13* Alsaeed 2025, KSA (Alsaeed et al., 2025)	Lung	36	F	SLE, APS	2021	INH, RIF, EMB, PZA INH, EMB, MOX	N/A	Cured
14* Alsaeed 2025, KSA (Alsaeed et al., 2025)	Lung	40	M	Smoking	2022	INH, RIF, EMB, PZA RIF, EMB, CLR	N/A	Cured
15* Alsaeed 2025, KSA (Alsaeed et al., 2025)	Lung	55	M	HIV, smoking	2023	INH, RIF, EMB, AZM	N/A	Improved
16* Alsaeed 2025, KSA (Alsaeed et al., 2025)	Bone	37	M	HIV	2023	INH, RIF, EMB, AZM RIF, EMB, MOX	N/A	Cured
17* Alsaeed 2025, KSA (Alsaeed et al., 2025)	Lung, Kidney	24	M	Gastrectomy	2024	INH, RIF, EMB, PZA RIF, EMB, AZM	N/A	Improved
18 Alsaeed 2025, KSA (Alsaeed et al., 2025)	Lung	28	F	None	2022	RIF, EMB, CLR	12	Cured
19 Garbati 2014, KSA (Garbati and Hakawi, 2014)	Lung	54	M	HIV	2014	INH, RIF, EMB, CLR	2	Cured
						RIF, EMB, CLR	10	
20 Al-Ammari 2016, KSA (Al-Ammari et al., 2016)	Lung, Lymphatic	30	M	HIV	2016	INH, EMB, PZA, MOX	N/A	Cured
21 Al-Dossary 2016, KSA (Al-Dossary et al., 2016)	Lung, Lymphatic	7	M	None	2016	RIF, EMB, CLR, CIP	2	Cured
						CLR, CIP	4	
22^¶^ Kosaraju 2024, KSA (Kosaraju et al., 2024)	Lung	67	M	Hx of TB	2024	RIF, EMB, AZM	6	Cured
						TG, AMK, LZD	12	
23 Almarhabi 2025, KSA (Almarhabi et al., 2025)	Bone, Lymphatic	39	F	None	2022	INH, RIF, EMB, CLR, MOX	4	Cured
						INH, RIF, EMB	8	
24 Abukhalid 2025, KSA (Abukhalid et al., 2025)	Joint	5	M	None	N/A	RIF, CLR	12	Cured
25 Abukhalid 2025, KSA (Abukhalid et al., 2025)	Lung, Liver, Bone	13	M	SCID Lymphoma	N/A	RIF, MOX, LZD	22	Cured
26 Abukhalid 2025, KSA (Abukhalid et al., 2025)	Skin, Lung, Lymphatic	2	M	AR-IMD91	N/A	RIF, EMB, CLR, CIP	24	Cured
27^#^ Habous 2025, UAE (Habous et al., 2025)	Lung	32	M	HIV	2025	RIF, EMB, MOX	N/A	Improved

Details of *M. riyadhense* cases with complete case descriptions (case reports/series). USA, United States of America; KSA, Kingdom of Saudi Arabia; UAE, United Arab Emirates; HIV, Human Immunodeficiency Virus; SLE, Systemic lupus erythematosus; APS, Antiphospholipid syndrome; Hx, history of; N/A, Information not available; SCID, Severe combined immunodeficiency secondary to deficiency of adenosine deaminase 2; AR-IMD91, Autosomal recessive immunodeficiency 91; INH, isoniazid; RIF, rifampin; EMB, ethambutol; PZA, pyrazinamide; MOX, moxifloxacin; CLR, clarithromycin; CIP, ciprofloxacin; AZM, Azithromycin; TG, Tigecycline; AMK, Amikacin; LZD, linezolid; LFX, levofloxacin; Sx, surgery.

*Cases 1 to 10 had susceptibility results available. See **Table 3**.

^†^Patient is a Saudi expatriate.

^‡^Patient who experienced relapse with CIP + CLR regimen.

^§^Patient originally from Germany, experienced 2 relapses while on INH + RIF.

^¶^Mycobacterium abscessus isolated only 2 months after the completion of RIF, EMB, and AZM. *Mycobacterium riyadhense* was not detected.

^#^Patient received ertapenem and azithromycin for eight weeks due to cooccurring non-typhoid *salmonella* psoas abscess.

## 3 Results

### 3.1 Search results

**Figure 1:**
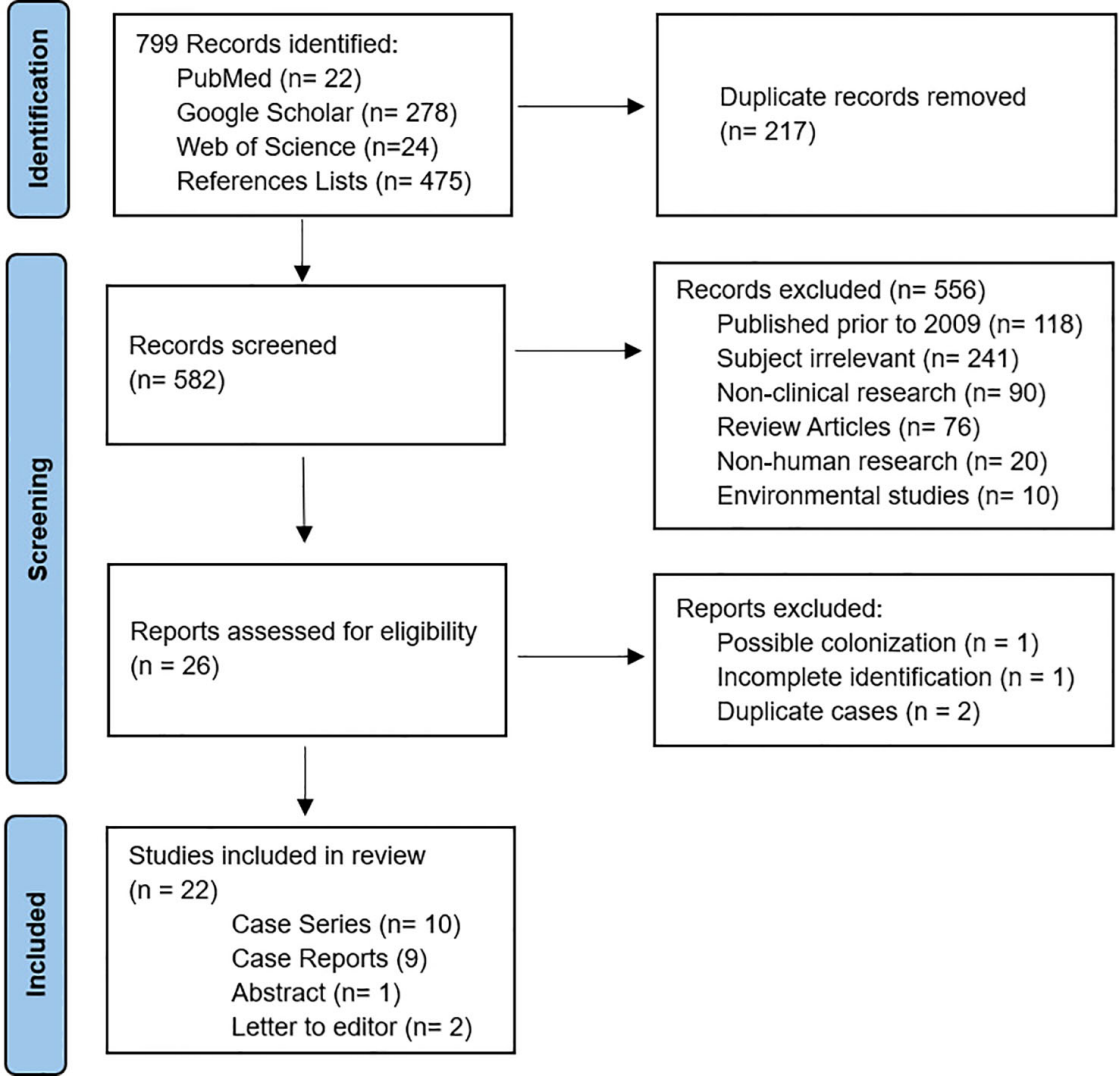
PRISMA flow-diagram of search results. Made using a downloadable template provided by the PRISMA guidelines website and published in the following citation: Page MJ, et al. BMJ 2021;372:n71. DOI: 10.1136/bmj.n71. (CC BY 4.0) license.

The initial search identified 324 articles (PubMed: 22, Web of Science: 24, Google Scholar: 278). In addition, 475 references were collected from included articles, totaling 799 articles for screening. Following duplicate removal and application of inclusion criteria, 26 articles were selected for full-text review. Four articles were excluded after full-text review, two of which did not provide details for the cases identified (Mohamed et al., 2016; Alabbas et al., 2023), as they were likely preliminary reports for cases reported later from the same centers (Alenazi et al., 2019; Abukhalid et al., 2025). One article was excluded due to uncertainty regarding the clinical relevance of *M. riyadhense*. The case was diagnosed by metagenomic sequencing of a terminal ileum biopsy with negative smear and culture results and co-occurrence of *M. smegmatis* and *M. kansasii* in an immunocompetent patient with Crohn’s disease (Hala et al., 2020). The fourth was excluded due to ambiguous identification with equal likelihood for *M. haemophilum* or *M. riyadhense* (Karri et al., 2021). Of note, two articles describing overlapping cases were retained, as each contributed complementary non-redundant data, and the information was merged (Guan et al., 2021; Almutairi et al., 2022). This resulted in 22 original research articles reporting on 69 cases being included for analysis (**Figure 1**).

### 3.2 Patient demographics and risk factors

**Figure 2:**
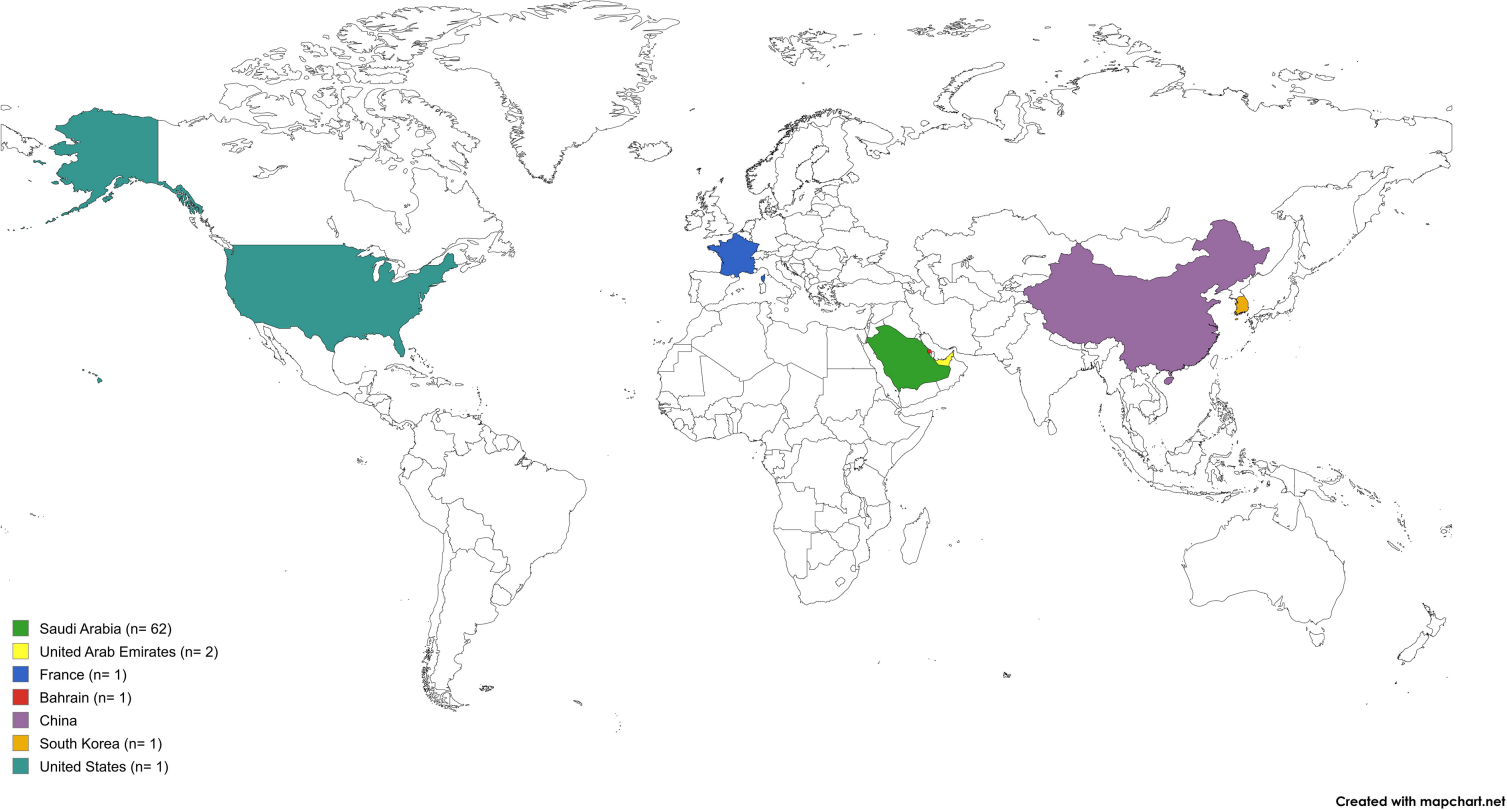
Global geographic distribution of reported *M. riyadhense* cases. Developed using Mapchart.net. CC BY-SA 4.0 license.

62 cases were reported from Saudi Arabia (van Ingen et al., 2009; Garbati and Hakawi, 2014; Saad et al., 2015; Al-Ammari et al., 2016; Al-Dossary et al., 2016; Varghese et al., 2017; Varghese et al., 2018; Alenazi et al., 2019; Varghese et al., 2019; Baadani et al., 2020; Almutairi et al., 2022; Kosaraju et al., 2024; Abukhalid et al., 2025; Almarhabi et al., 2025; Alsaeed et al., 2025), two from the United Arab Emirates (Sawan et al; Habous et al., 2025), and five individual cases were reported from France (Godreuil et al., 2012), Bahrain (Godreuil et al., 2012), China (Xu et al., 2017), South Korea (Choi et al., 2012), and the United States (Henderson et al., 2012) (**Table 1**, **2**). Most patients were middle-aged, with a median of 35.5 years (IQR 22.7–48 years) (range 2–82 years), and the majority being male (77%, n= 41/53). Six patients were elderly and nine were pediatrics. **Figure 2** shows a global map of reported cases of *M. riyadhense*.

Risk factors were reported in 24 cases, with immunodeficiency being predominant (n= 17). Other risk factors were history of tuberculosis (n= 2), malignant lymphoma (n= 2), gastric surgery (n= 2), smoking (n= 2), systemic lupus (n= 1), cystic fibrosis (n= 1), and trauma (n= 1). Among the causes of immunodeficiency, human immunodeficiency virus (HIV) was the major contributor (n= 14) (Garbati and Hakawi, 2014; Al-Ammari et al., 2016; Alenazi et al., 2019; Baadani et al., 2020; Almutairi et al., 2022), with three remaining patients having congenital neutropenia (CN) (Varghese et al., 2019), autosomal recessive immunodeficiency 91 (AR-IMD91) (Abukhalid et al., 2025), or severe combined immunodeficiency (SCID) (Abukhalid et al., 2025). Four immunodeficient patients had cooccurring risk factors, including a patient with HIV who had a history of tuberculosis and another who was a smoker (Kosaraju et al., 2024; Alsaeed et al., 2025). In two other patients malignant lymphoma cooccurred with either HIV or SCID (**Appendix A**) (Abukhalid et al., 2025). CD4 counts were provided for eight out of 14 patients with HIV. The majority (n= 6/8) exhibited counts below 50 cells/mm³ (Garbati and Hakawi, 2014; Alenazi et al., 2019; Habous et al., 2025), (**Appendix A**). Two remaining patients had counts above 200 cells/mm³ at the time of infection (Al-Ammari et al., 2016), (**Appendix A**). Out of seven cases reported in countries other than Saudi Arabia, one case reported by Henderson et al. involved a Saudi expatriate residing in the United States (Henderson et al., 2012). Travel to Saudi Arabia was not reported for the remaining six cases and contact with a patient infected with *M. riyadhense* was not reported for any case.

### 3.3 Clinical manifestations

Pulmonary disease was the most common clinical presentation, being observed in 52 out of 68 cases with a reported clinical manifestation (76%, 52/68). Extrapulmonary infection occurred in 33% of patients (23/68), with lymphadenitis being most common (19%, 13/68), followed by osteomyelitis (11%, 8/68), glomerulonephritis (n= 1), and septic arthritis (n= 1). Disseminated infection occurred in eight patients, most of whom presented with mixed pulmonary and extrapulmonary disease (n= 7). In addition to pulmonary involvement, the extrapulmonary focus was lymphadenitis (n= 3), pleural effusion (n= 1), glomerulonephritis (n= 1), combination of hepatic and bone (n= 1, a child with SCID), and combination of lymph node and cutaneous involvement (n= 1, a child with AR-IMD91). The only patient with disseminated infection without pulmonary involvement had lymphadenitis and osteomyelitis (Almarhabi et al., 2025). Among 16 patients with exclusive extrapulmonary disease, the majority had lymphadenitis (n= 8), followed by osteomyelitis (n= 5) and septic arthritis (n= 1). Of note, two progressive extrapulmonary infections presented as sinusitis progressing to osteomyelitis and optic neuritis (van Ingen et al., 2009), and frontal bone osteomyelitis progressing to dura mater and cerebral surface involvement (Saad et al., 2015).

Age-specific manifestations varied considerably. Pediatric patients more often exhibited lymph node involvement (n= 5), with isolated lymphadenitis being most common (n= 3) (Varghese et al., 2019). Lymphadenitis cooccurred with pulmonary infection in two pediatric patients without risk factors. Only one child with congenital neutropenia presented with isolated pulmonary involvement (Varghese et al., 2019), and two other immunocompromised children each had involvement of three organs, as described earlier (Abukhalid et al., 2025). In contrast, all elderly patients (n= 6) and all but two patients with HIV (n= 12/14) had pulmonary involvement, typically in the isolated form (n= 16). One patient with HIV presenting with pulmonary infection developed lymphadenitis after starting antiretrovirals (Al-Ammari et al., 2016). Of the patients with HIV without pulmonary involvement (n= 2), one had isolated osteomyelitis (Alsaeed et al., 2025) and the other case lacked any clinical details (Baadani et al., 2020).

### 3.4 Identification and antimicrobial susceptibility

Identification methods were reported in 50 cases. In the majority (82%, 41/50), microbiological identification relied on Sanger sequencing of gene regions such as 16S, ITS, rpoB, or HSP65. Metagenomic sequencing was utilized in nine cases (Guan et al., 2021; Almutairi et al., 2022; Almarhabi et al., 2025). Susceptibility testing was conducted on 17 isolates (**Table 3**). Methods included broth microdilution (n= 4), agar dilution (n= 1), Bactec MGIT system (n= 1), and broth microdilution with absolute concentration (n= 1). Testing methodology was unspecified for ten isolates. Breakpoints and interpretative standards were reported for only three isolates; two utilized CLSI M24-A (2003) (Godreuil et al., 2012) and one applied CLSI M24-A2 2nd edition (2011) (Choi et al., 2012).

**Table 3:** Results of antimicrobial susceptibility data for *M. riyadhense*; 10 reported isolates.

Drug; # of tested isolates	Case # interpretation; MIC (ug/ml)																	Susceptible%
	1	2*	3*	4^†^	5	6^‡^	7^§^	8^#^	9	10	11	12	13	14	15	16	17	
Isoniazid; 8	I; 0.5	S; 0.5	S; 0.25	I; 1	R; 1	R; 0.4	R; 0.2^§^	S										37.5%
Rifampin; 17	S; < 0.5	S;< 0.12	S;< 0.12	S; 0.2	S; 1	S; 1	S; 0.25	S	S	S	S	S	S	S	S	S	S	100%
Ethambutol; 11	S; < 2	S; < 0.5	S; < 0.5	S; 5	S; 7.5	S; 5	S;< 0.25	S	S		S	S						100%
Ethionamide; 6	S; < 1	S; 0.3	S; 0.3	S; < 1	S; 10		S; 40^§^											100%
Moxifloxacin; 5	S; < 0.5	S;< 0.12	S; < 0.12				S; 0.5					S						100%
Azithromycin; 6	S; < 32												S	S	S	S	S	100%
Clarithromycin; 16	S; < 4	S; 0.12	S; 0.12	S; < 2	S; < 4	I; 16^¶^	S; < 0.5	S	S	S	S		S	S	S	S	S	94%
Streptomycin; 7	S; < 2	S; 1	S; < 0.5	S; 5	S; 2	S; 1	R; 10^§^											85.7%
Amikacin; 7	S; < 2	S; < 1	S; < 1	R; 10	S; 6		S; < 1					S						85.7%
Rifabutin; 6	I; 0.25	S; <0.25	S; < 0.25	S; < 2			S; 20^§^					S						83.3%
Cycloserine; 4	R; 16			S; 20	S; 60		S; 30^§^											75%
Ciprofloxacin; 8	S; < 1	S; 1	S; 0.12	S; 2	S; < 1		I; 2	I				S						75%
Doxycycline; 6	R; > 16	R; 16	R; 4		I; 8		S; 1	I										16.6%
SMX/TMP; 6	S; 9.5/0	N; 2.3/<0.1	N; 38/< 2				S; 8/-	R				S						
Kanamycin; 3	R; 8				R; 6		S; 40^§^											
PAS; 3				R; > 1	R; 8		R; 1^§^											
Linezolid; 3		S; < 1	S; < 1									S						
Capreomycin; 2					S; 10		S; 40^§^											
Clofazimine; 2	S;< 0.06			S;< 0.5														
Levofloxacin; 2	R; 4						R; 2^§^											
Imipenem; 2					R; >16		N; 32											
Pyrazinamide; 2						R; 100		S										
Ofloxacin; 1							R; 2^§^											
Tobramycin; 1		N; 2					N; 2											
Cefoxitin; 1		N; 128					N; 128											

S, sensitive; I, intermediate; R, resistant; N, no interpretation; MIC, minimum inhibitory concentration (ug/ml); SMX/TMP, Sulfamethoxazole/Trimethoprim (results for each separated by/); PAS, para-amino salicylate.

*breakpoints are based on CLSI M24-A (2003).

^†^susceptibility was tested using agar dilution instead of broth-microdilution.

^‡^tested using the Bactec MGIT system.

^§^For some antibiotics the absolute concentration method was used instead of broth microdilution. Broth microdilution MICs are used when available and are not labeled with superscript. Breakpoints are based on CLSI M24-A2 2nd e.d (2011).

^¶^tested using the E-test method.

^#^this isolate was tested once before and twice after starting antibiotics. The final sensitivity results are provided.

No MICs are available for cases # 8 to 17.

The source for the interpretation was only provided for cases # 2, 3, and 7.

Susceptibility % = (S/S + I + R) x 100, Given if at least 4 results exist.

Rifampin and Ethambutol demonstrated universal *in vitro* susceptibility across all tested isolates, (17/17 and 11/11 respectively). Likewise, Ethionamide, Azithromycin, and Moxifloxacin exhibited 100% susceptibility, although with fewer tested isolates (6/6, 6/6, and 5/5 respectively). In contrast, Isoniazid showed susceptibility in only 3 out of 8 tested isolates, two of which were interpreted using CLSI M24-A (2003), while the interpretive methodology was unspecified for the third.

Emerging resistance during therapy was analyzed in two isolates (Sawan et al; Henderson et al., 2012). The first was from a patient who received three months empirical monotherapy with levofloxacin, initially showing clinical improvement followed by relapse; subsequent testing revealed levofloxacin resistance (Henderson et al., 2012). The other isolate was tested before starting therapy and twice after starting Isoniazid, Rifampin, Ethambutol, and Pyrazinamide (Sawan et al). Initial testing showed sensitivity to all 1st-line and 2nd-line anti-TB agents, later results revealed emerging resistance to Clarithromycin, Doxycycline, Trimethoprim-Sulfamethoxazole, and Ciprofloxacin. In the third test, susceptibility to Clarithromycin was redemonstrated, with resistance to the other agents maintained. Susceptibility to Isoniazid, Rifampin, Ethambutol, and Pyrazinamide were retained across all repeated tests, although MIC values were not reported. This was in spite of treatment failure occurring twice with Isoniazid + Rifampin in this patient (Sawan et al).

### 3.5 Treatment strategies and outcomes

Therapeutic approaches were reported for 39 patients: 12 patients from a retrospective study with incomplete case details (Varghese et al., 2017) and 27 case reports/series with mostly complete information, which are summarized in **Table 2**. The majority (n= 26) responded to the initial treatment regimen, while three pulmonary cases required a change in therapy (**Table 2**). Response to treatment was not mentioned for two patients who were on Isoniazid + Rifampin (Varghese et al., 2017), one of whom passed away with undisclosed circumstances (Varghese et al., 2017). The first line anti-TB agents were most frequently utilized; Rifampin (n= 28), Isoniazid (n= 23), Ethambutol (n= 19). These were followed by Macrolides (n= 15); Clarithromycin (n= 13), Azithromycin (n= 2), and Fluroquinolones (n= 11); Moxifloxacin (n= 8), Ciprofloxacin (n= 3), Levofloxacin (n= 1).

Eight patients followed a step-wise induction-to-consolidation approach, whereas 19 patients received the same regimen throughout treatment. A combination that included Rifampin and Ethambutol was administered throughout the entire treatment course in 11 cases (28%, 11/39). 15 cases received a 2–3 drug regimen, mostly incorporating Rifampin (n= 13) and as part of a triple therapy combination with Rifampin, Isoniazid, and Clarithromycin (n= 5). Four patients switched antibiotic combinations during their course. Three were due to failure of the initial regimen (**Table 2**; cases# 1, 3, 8), and one was due to coinfection with *M. abscessus* that occurred after culture clearance of *M. riyadhense* (**Table 2**; case# 14). Failed regimens included Levofloxacin empirical monotherapy (Henderson et al., 2012), Clarithromycin + Ciprofloxacin (Godreuil et al., 2012), and during Isoniazid + Rifampin consolidation (occurring twice in a single case) (Sawan et al). The two former cases achieved cure after the introduction of all 4 anti-TB agents, while the latter ultimately required surgical intervention. The final regimen for this patient included Moxifloxacin, Linezolid, and Clarithromycin in addition to the first line anti-TB agents, however, final treatment and follow up durations were not mentioned (Sawan et al). Failures occurred in spite of confirmed susceptibility to both antibiotics in the dual therapy regimens (Sawan et al; Godreuil et al., 2012).

Treatment durations could be assessed in 16 cases (**Table 2**). The durations varied between cases, mainly depending on the patient’s characteristics and type of disease. A longer duration was used in cases with pulmonary involvement; median 14 months (range: 6–24 months), than in cases without pulmonary involvement; median 12.5 months (range: 9–15 months). Cases of disseminated infection had the longest duration of therapy; median 17 months (range: 6–24 months). Durations were similar between immunocompromised patients; median 12 months (range: 10–24 months), and immunocompetent patients; median 13 months (range: 6–20 months). However, two patients with hereditary immunodeficiency received a longer duration of 22 and 24 months, respectively (Abukhalid et al., 2025). Adverse effects were reported in two patients treated with Moxifloxacin, one experienced minor dyspepsia while the other developed severe tendinitis requiring discontinuation (Sawan et al; Almarhabi et al., 2025).

### 3.6 Quality assessment

Of eleven case reports, seven had a complete score of 8/8 (van Ingen et al., 2009; Choi et al., 2012; Henderson et al., 2012; Garbati and Hakawi, 2014; Al-Dossary et al., 2016; Kosaraju et al., 2024; Almarhabi et al., 2025), three had a score of 6/8 due to missing information regarding treatment and follow up (Sawan et al; Al-Ammari et al., 2016; Habous et al., 2025), and one had a score of 2/8 due to multiple missing information as outlined in **Table 1**, **2** (Xu et al., 2017). Scores for the eleven case series articles were 10/10 for four articles (Godreuil et al., 2012; Saad et al., 2015; Alenazi et al., 2019; Abukhalid et al., 2025), 8/10 for five articles due to missing clinical and follow up information (Varghese et al., 2017; Varghese et al., 2019; Guan et al., 2021; Almutairi et al., 2022; Alsaeed et al., 2025), and 7/10 for two articles due to missing information for patient demographics, clinical data, and follow up data (Varghese et al., 2018; Baadani et al., 2020).

## 4 Discussion

Since the identification of *M. riyadhense*, sixteen years ago, it has emerged as a global cause of infection in individuals both with and without predisposing factors. Although a relatively high number of cases have been reported from Saudi Arabia, travel to the country has not been identified as a risk factor for developing infection, nor has contact with people who are infected (Sawan et al; Choi et al., 2012; Godreuil et al., 2012; Henderson et al., 2012; Xu et al., 2017). It is more likely that, similar to other NTM species, *M. riyadhense* infections originate from contact with the environment, where this pathogen has been demonstrated to exist in several countries (Londono Zuluaga, 2016; Hongslo and Jansson, 2016; Maidin et al., 2016; King et al., 2017; Narendrula-Kotha and Nkongolo, 2017; Alqumber, 2021; Rahmatika et al., 2022). *M. riyadhense* may also have the capacity to colonize human tissues, as suggested by a case excluded from this review, in which it was detected in the gastrointestinal tract of a patient with Crohn’s disease alongside two other NTM species (Hala et al., 2020). In this review, we demonstrated that *M. riyadhense* infections present with a wide range of clinical features that may be indistinguishable from MTB or other NTM infections. We also emphasized that susceptibility results, which were extrapolated from breakpoints for other slow-growing mycobacteria (Choi et al., 2012; Godreuil et al., 2012), do not consistently predict response to treatment (Sawan et al; Godreuil et al., 2012).

We believe that cases summarized in this review, likely underestimate the true incidence of *Mycobacterium riyadhense* for several reasons. The most significant of which is the lack of affordable methods that can distinguish *M. riyadhense* from TB and other NTM species (Almarhabi et al., 2025). Additionally, in TB-endemic areas, there is a common practice of initiating empiric anti-TB treatment before obtaining cultures (Agashe et al., 2020), which could complicate the identification of *M. riyadhense*, due to its similar presentation and susceptibility to first-line anti-TB agents. This factor has contributed to delaying the diagnosis of *M. riyadhense* in a previously reported case (Al-Ammari et al., 2016). Widespread adoption and validation of advanced diagnostic tools is necessary to precisely understand the epidemiology of emerging pathogens such as *M. riyadhense*.

While most therapeutic choices were empirically chosen, the limited susceptibility data available for *M. riyadhense* suggests consistent susceptibility to Rifampin (17/17), Ethambutol (11/11), Azithromycin (6/6), Ethionamide (6/6), and Moxifloxacin (5/5). Notably, the majority of tested isolates had isoniazid resistance or intermediate susceptibility (n= 5/8, 62%), which was distributed between cases in Saudi Arabia (van Ingen et al., 2009; Saad et al., 2015), Korea (Choi et al., 2012), and the United States (Saudi expatriate) (Henderson et al., 2012). It should be reemphasized, however, that susceptibility results did not always correlate with successful treatment outcomes (Sawan et al; Godreuil et al., 2012). For example, one case treated with Clarithromycin + Ciprofloxacin resulted in failure, despite the isolate having the lowest minimum inhibitory concentration (MIC) for both agents (0.12 ug/ml for both) before starting therapy (Godreuil et al., 2012). In another case, resistance to Clarithromycin and Ciprofloxacin as well as to other agents emerged after therapy, while the agents that failed to achieve cure (Isoniazid + Rifampin) remained susceptible in two repeated tests after therapy (Sawan et al).

Although all failed regimens were either monotherapy or dual therapy, this does not necessarily indicate that dual therapy is ineffective for *M. riyadhense*, as it has been used successfully in other cases, including Isoniazid + Rifampin (Varghese et al., 2017) and Clarithromycin + Ciprofloxacin (Al-Dossary et al., 2016). Other successful dual agent combinations included Moxifloxacin + Clarithromycin (Alenazi et al., 2019) as well as Rifampin + Clarithromycin (Abukhalid et al., 2025).

Another inconclusive subject is the optimal duration of therapy for *M. riyadhense*. A 12-month course was most commonly used across both pulmonary and extrapulmonary cases (31%, 5/16). However, a shorter duration might be effective and is certainly desirable. For instance, 6 months was sufficient in a pediatric patient with disseminated infection (Al-Dossary et al., 2016), 9 months was effective in an adult with extrapulmonary disease (van Ingen et al., 2009), and 10 months led to cure in an adult with HIV and pulmonary infection (Alenazi et al., 2019), all without reported relapse (**Table 2**). A longer duration may be more favorable in immunocompromised patients, especially if a return to normal immune function is not expected.

Despite illustrating these important findings, this review had significant limitations. Firstly, most of the available data comes from case reports or retrospective studies, many of which lacked complete clinical or microbiological details. Moreover, the geographic distribution of reports remains skewed, with the majority originating from Saudi Arabia, despite evidence of global pathogen presence. Additionally, reliance on published literature means that included cases will be biased towards countries and institutions with access to means of publication and the costly identification methods. Finally, susceptibility testing was reported in only 17 out of 69 cases, and the specific testing methodologies were often not disclosed.

In summary, *M. riyadhense* has become a notable global pathogen that presents a significant diagnostic and therapeutic challenge, especially due to the lack of reliable diagnostic methods to differentiate it from MTB and other non-tuberculous mycobacteria. This misidentification can lead to inappropriate treatment and delayed patient care. Although several antibiotics demonstrate *in vitro* susceptibility rates exceeding 80%, clinical outcomes remain inconsistent, highlighting the need for individualized treatment guided by thorough analysis of each patient’s history and risk factors, as well as the type of infection and antimicrobial susceptibility. The current evidence is limited, primarily consisting of case reports, which underscores the need for more extensive studies to better understand the epidemiology, optimal treatment regimens, and long-term outcomes associated with *M. riyadhense* infections. Addressing these gaps is crucial for improving clinical practice and patient outcomes.

## Data Availability

The original contributions presented in the study are included in the article/**Supplementary Material**. Further inquiries can be directed to the corresponding author.
